# Human-Induced CD49a^+^ NK Cells Promote Fetal Growth

**DOI:** 10.3389/fimmu.2022.821542

**Published:** 2022-02-04

**Authors:** Xianghui Du, Huaiping Zhu, Defeng Jiao, Zhigang Nian, Jinghe Zhang, Yonggang Zhou, Xiaohu Zheng, Xianhong Tong, Haiming Wei, Binqing Fu

**Affiliations:** ^1^The Department of Obstetrics and Gynecology, First Affiliated Hospital of University of Science and Technology of China, Division of Life Sciences and Medicine, University of Science and Technology of China, Hefei, China; ^2^The CAS Key Laboratory of Innate Immunity and Chronic Disease, School of Basic Medical Sciences, Division of Life Sciences and Medicine, University of Science and Technology of China, Hefei, China; ^3^Institute of Immunology, University of Science and Technology of China, Hefei, China; ^4^The Section of Experimental Hematology, First Affiliated Hospital of University of Science and Technology of China, Division of Life Sciences and Medicine, University of Science and Technology of China, Hefei, China

**Keywords:** maternal-fetal interface, decidual tissue-resident NK cells, CD49a, low cytotoxic, fetal growth

## Abstract

CD49a^+^ natural killer (NK) cells play a critical role in promoting fetal development and maintaining immune tolerance at the maternal-fetal interface during the early stages of pregnancy. However, given their residency in human tissue, thorough studies and clinical applications are difficult to perform. It is still unclear as to how functional human CD49a^+^ NK cells can be induced to benefit pregnancy outcomes. In this study, we established three no-feeder cell induction systems to induce human CD49a^+^ NK cells from umbilical cord blood hematopoietic stem cells (HSCs), bone marrow HSCs, and peripheral blood NK cells *in vitro*. These induced NK cells (iNKs) from three cell induction systems display high levels of CD49a, CD9, CD39, CD151 expression, low levels of CD16 expression, and no obvious cytotoxic capability. They are phenotypically and functionally similar to decidual NK cells. Furthermore, these iNKs display a high expression of growth-promoting factors and proangiogenic factors and can promote fetal growth and improve uterine artery blood flow in a murine pregnancy model *in vivo*. This research demonstrates the ability of human-induced CD49a^+^ NK cells to promote fetal growth *via* three cell induction systems, which could eventually be used to treat patients experiencing adverse pregnancy outcomes.

## Introduction

During the early stages of pregnancy, the endometrium is transformed into decidual tissue by both estrogen and progesterone, forming a maternal-fetal interface as a result of the interaction between mother and fetus (1). During the first trimester, CD45^+^ leukocytes account for up to 40% of the total number of cells at the maternal-fetal interface, in which >70% of all leukocytes in human decidua are natural killer (NK) cells (2, 3). These human decidual NK cells (dNKs) have distinct functional and phenotypic characteristics including CD56^bright^CD16^-^ phenotype (4), poor cytotoxicity with high expression of inhibitory receptors and low expression of cytotoxicity-activating receptors (5–7). Previous research has demonstrated that dNKs can maintain immune tolerance at the fetal-maternal interface (8), promote decidual vascularization, spiral artery formation and extravillous trophoblast invasion by a non-cytotoxic mechanism (9–12), enhance fetal growth during the critical early stages of fetal development (13), and contribute to placenta formation (14). Abnormalities in NK cells, which include lower cell counts or dysfunction, can negatively affect the balance of the fetal-maternal microenvironment, and eventually cause pathological pregnancy (15–17).

CD49a is an integrin alpha subunit that binds collagen and laminin and has been confirmed as a marker to identify tissue-resident NK (trNK) cell subsets in mice (18, 19). Our group found that uterine NK cells (uNKs) in humans have a large amount of CD49a^+^ trNK cells, specifically, CD49a^+^Eomes^+^ uterine trNK cells, which comprise 85% of all NK cells from normal human decidua during the first trimester, secrete growth-promoting factors, and enhance fetal growth during the critical early stages of fetal development (13). Decreased CD49a^+^Eomes^+^ uterine trNK cells and the secretion of impaired growth-promoting factors are both present in miscarriage patients (13). However, uterine CD49a^+^ trNK subsets in menstrual blood also can predict the abnormal endometrial status (20). As such, the therapeutic administration of CD49a^+^ NK cells for human pregnancy-associated disorder could be able to mitigate the effects of restricted nourishment within the uterine microenvironment.

Several published reports have demonstrated that CD34^+^ hematopoietic stem cells (HSCs) can differentiate into NK cells (21) through a range of transcription factors and extrinsic signals from multiple cytokines (22). The fms-like tyrosine kinase 3 ligand (Flt3L) and stem cell factor (SCF) promote the proliferation and differentiation of HSCs *via* common lymphoid progenitors (CLPs) by interacting with their receptors on the cell surface (23). Interleukin-15 (IL-15) promotes the development of NK lineage-restricted progenitors to mature NK cells and the proliferation of NK cells (24). In particular, TGF-β promotes the conversion of CD16^+^ peripheral blood NK cells (pNKs) into CD16^-^ NK cells (25). Hypoxia affects the function of NK cell immune-regulation and migration, including upregulating VEGFA and CXCL8 (26). These cytokines or hypoxia conditions could yield cells similar to dNK cells (27, 28). However, it is unclear whether these platforms support the generation of CD49a^+^ human decidual-like NK cells, which have a functional distinguished signature and can promote fetal growth.

To bypass these limitations, we investigated three *in vitro* platforms supporting the generation of human cytokine-induced CD49a^+^ NK cells, which are phenotypically and functionally similar to functional dNKs.

## Materials and Methods

### Human Sample

Fresh umbilical cord blood, bone marrow, and decidual samples were obtained at the First Affiliated Hospital of the University of Science and Technology of China (Hefei, China). Umbilical cord blood was collected in heparin-coated collection bags from healthy donors delivering at term (maternal age range: 21-36 years). Bone marrow samples were obtained from patients with diagnostic bone marrow puncture, whose clinical diagnoses shown their HSCs having no appreciable functional difference with healthy people. First-trimester decidual samples (5-9 weeks) were obtained from voluntarily-terminated pregnancies (age range: 22-40 years). The terminations were for reasons unrelated to the pathology of pregnancy and fetal abnormality. Normal peripheral blood mononuclear cells (PBMCs) were obtained at the Blood Center of Anhui Province (Hefei, China). Informed consent was obtained from each donor. All human samples used in the present study were obtained under the approval of the Ethics Committee of the University of Science and Technology of China (USTCEC201800001; Hefei, China).

### Mice

Immunodeficient NCG (NOD/ShiLtJGpt-*Prkdc^em26Cd52^Il^2rgem26Cd22^*/Gpt) mice were purchased from GemPharmatech Co., Ltd., Nanjing, China. All mice were maintained under pathogen-free conditions. All experimental procedures involving mice followed the National Guidelines for Animal Usage in Research (China) and were approved by the Ethics Committee of the University of Science and Technology of China (Reference NO. USTCACUC1801018).

### Human Sample Isolation

Umbilical cord blood was collected immediately after delivery in heparin-coated collection bags (approximately 30 mL) and isolated using Ficoll density gradients (LTS1077, TBD, Tianjin, China) in 50-mL tubes. Fresh bone marrow samples (approximately 2-3 mL) were freshly collected in a heparin tube from outpatient bone marrow aspiration and isolated using Ficoll density gradients in a 15-mL tube. CD34^+^ hematopoietic stem cells (HSCs) were separated using a CD34^+ ^selection kit (130-046-702, Miltenyi Biotec, Germany) from mononuclear cells of umbilical cord blood and bone marrow. Decidual mononuclear cells were extracted from decidual samples after digestion with collagenase type IV (C-5138, Sigma). Stromal cells were then removed after the adherents were cultured. Peripheral blood mononuclear cells (PBMC) were separated over Ficoll density gradients in 50-mL tubes. NK cells were purified *via* negative selection with a magnetic-activated cell sorter (MACS) kit (130-092-657, Miltenyi Biotec, Germany).

### Cell Culture and Induce

The iNK cells were cultured in GMP Serum-free Stem Cell Growth Medium (20802, CellGenix) supplemented with 10% fetal bovine serum (FBS, 10091, Gibco). The cells were cultured at an initial density of 1×10^6^ cells/mL. Cord blood CD34^+^ HSCs were usually cultured in 24-well cell culture plates at the beginning, while bone marrow CD34^+^ HSCs were cultured in 48- or 96-well cell culture plates and pNK cells were in 25T flasks. For CB-iNK cells and BM-iNK cells, SCF (20 ng/mL, AF−300−07, PeproTech), Flt3L (30 ng/mL, AF−300−19, PeproTech) were added to the medium during the first two weeks, and IL-15 (20 ng/mL, AF−200−15, PeproTech) was added on day 4 of culturing. At the end of day 14, the dosage of Flt3L and SCF gradually decreased until it was no longer added, while afterward only IL-15(30 ng/mL) was added from day 21. Cells were induced and cultured for approximately five weeks at 37°C in a humid atmosphere with 5% CO_2_. Two times a week, half of the medium was removed and replaced with fresh medium and cytokines. For the pNK-iNK group, TGF-β1 (5 ng/mL, AF−100−21C, PeproTech), hCG (10 IU/mL, LIVZON Group, China), and IL-15 (10 ng/mL) were added to the medium. Equivalent fresh medium was added after three or four days of culture, after which the plates were incubated under 1-2% O_2_ for the next 4~6 days.

### Flow Cytometry Assay

Cell suspensions were surface-labeled for human antibodies at 4°C for 30 min in the dark, washed twice with PBS, and then tested. For intracellular staining of pleiotrophin and osteoglycin, the cells were cultured for 4 h in the presence of monensin (2.5 μg/mL; Sigma) and then collected for treatment with Permeabilization Buffer from the Foxp3 Transcription Factor Staining Buffer Set (00-5523-00, eBioscience), according to the manufacturer’s instructions. Flow cytometry was performed on a BD LSRII and analyzed by FlowJo. All antibodies for flow cytometry staining are displayed in **Table S1**.

### RNA-seq

NK cells were washed with PBS three times and lysed using Trizol Reagent (Invitrogen). The samples were then sent to Gene Denovo Biotechnology Co. (Guangzhou, China) on dry ice. There were four cases in each group and n=20 donors. RNA quality was assessed with an Agilent 2100 Bioanalyzer (Agilent Technologies, Palo Alto, CA, USA) and checked using RNase-free agarose gel electrophoresis. The enriched mRNA was fragmented into short fragments using fragmentation buffer and reverse transcripted into cDNA. The cDNA fragments were then purified with QiaQuick PCR extraction kit (Qiagen), end-repaired, a base was added, and they were ligated to Illumina sequencing adapters. The ligation products were size-selected *via* agarose gel electrophoresis, PCR amplified, and sequenced using Illumina Novaseq6000. To obtain clean, high-quality reads, the reads were further filtered by fastp (version 0.18.0). We built an index of the reference genome, and paired-end clean reads were mapped to the reference genome using HISAT2.2.4 with “-rna-strandness RF” and other parameters set as a default. The mapped reads of each sample were assembled using StringTie v1.3.1 in a reference-based approach. For each transcription region, an FPKM (fragment per kilobase of transcript per million mapped reads) value was calculated to quantify its expression abundance and variations using RSEM software. RNA differential expression analysis was performed using DESeq2 software between two different groups. The genes/transcripts with p-value<0.05 and absolute fold change>2 were considered differentially expressed genes/transcripts. We performed gene set enrichment analysis using GSEA and MSigDB software programs to identify whether a set of genes in specific KEGG pathways show significant differences in the two groups. We briefly implemented the gene expression matrix and ranked the genes using the SignaltoNoise normalization method. Enrichment scores and p-value were calculated in default parameters.

### Immunofluorescence

NK cells were incubated with anti-human NCAM antibodies (3576S, CST, 1:100 dilution) and fluorescence-conjugated secondary antibodies (A-11029, Invitrogen, 1:200 dilution) in PBS (with 5% goat serum). The NK cells were then combined with Fixation/Permeabilization (00-5123, Invitrogen) and the Permeabilization Buffer (00-8333, Invitrogen) to complete the final washing step and cell resuspension. The NK cells were then incubated with primary antibodies and fluorescence conjugated secondary antibodies (A-11035, Invitrogen, 1:200 dilution). The primary antibodies include Rabbit mAb IgG Isotype control (3900S, CST, 1:50 dilution), Rabbit anti−Human Pleiotrophin Antibody (LS-C162291, Lifespan, 1:50 dilution), and Rabbit anti−Human Osteoglycin Antibody (LS-B10948, Lifespan, 1:50 dilution). All antibodies were incubated at room temperature for 1 hour. The nucleus was finally stained with DAPI. Samples were imaged using an LSM 880 + Airyscan system (Zeiss).

### Carboxy Fluorescein Succinimidyl Ester–Based Cytotoxicity Assay

The human leukemic cell line, K562, was purchased from the Shanghai Cell Bank (Chinese Academy of Sciences, Shanghai, China), and used as target cells. The cytotoxic activity of NK cells was determined with a carboxy fluorescein succinimidyl ester (CFSE)-based cytotoxicity assay (29). The target cells were briefly labeled with 5 μM CFSE (C34554, Invitrogen) for 15 min at 37°C. Effector cells (NK cells) and target cells (K562) were added to 96-well plates at different effector-target ratios for 5 hours at 37°C and 5% CO_2_. The cells were cultured in RPMI-1640 (SH30027.01, Hyclone) containing 10% FBS (10091, Gibco). To detect dead cells, 7-AAD (555816, BD Pharmingen) was added and the samples were directly analyzed using flow cytometry. The percentage of cytotoxicity was calculated as follows: % cytotoxicity = 100 × (experimental release - spontaneous release)/(100 - spontaneous release).

### Adoptive Transfer of NK Cells

NCG females were randomly mated with males, while the detection timing of a copulation plug was regarded as gd0.5. The NK cells were suspended in 200 μL of PBS and injected *via* the tail vein into pregnant females at gd6.5 and gd10.5 (2×10^6^ cells/mouse). Similar amounts of PBS were injected as a control. At gd16.5, the pregnant mice were euthanized. The weight and length of the live fetus and the weight and diameter of the corresponding placenta were recorded from each group.

### Doppler Ultrasound Imaging

A Vinno 6 performance real-time ultrasound scanner (Vinno Corporation, Suzhou, China) was used for ultrasound measurements. The pregnant mice were anesthetized *via* intraperitoneal injection of 2% pentobarbital sodium and positioned on a warm platform to maintain euthermia. The abdominal hair was removed with a depilatory agent. Pre-warmed ultrasound gel was applied to the depilated abdomen skin, while the bladder was used as a reference point to move the transducer to the abdomen and trace implantations. B-Mode was used to measure implantation areas at gd6.5 and placental conditions (thickness, diameter, and area) at gd12.5 in a 2D grayscale image. CF-Mode was used to visualize blood flow in the maternal uterine artery (UA) and fetal umbilical artery (UmA) at gd12.5. The pulsed wave doppler sample volume was adjusted and subtle positional changes of the transducer were made to obtain the blood flow parameters as close to parallel flow as possible. PW-Mode was used to quantify blood flow through the vessels. Peak systolic velocities (PSVs) and end-diastolic velocities (EDVs) of UAs and UmAs were recorded. The software automatically calculated the resistance index (RI). RI= (PSV - EDV)/PSV.

### Statistical Analyses

Statistical significance was determined *via* GraphPad version 8. Statistical significance between the two groups was identified *via* unpaired two-tailed t-tests, and between multiple groups by one-way analysis of variance (ANOVA). Data represent means ± SD or mean ± SEM. p < 0.05 was considered statistically significant (* p < 0.05; ** p < 0.01; *** p < 0.005; **** p < 0.0001; ns, not significant).

## Results

### Induced CD49a^+^ NK Cells Can Be Efficiently Generated From Three Different Sources *In Vitro*

**Figure 1:**
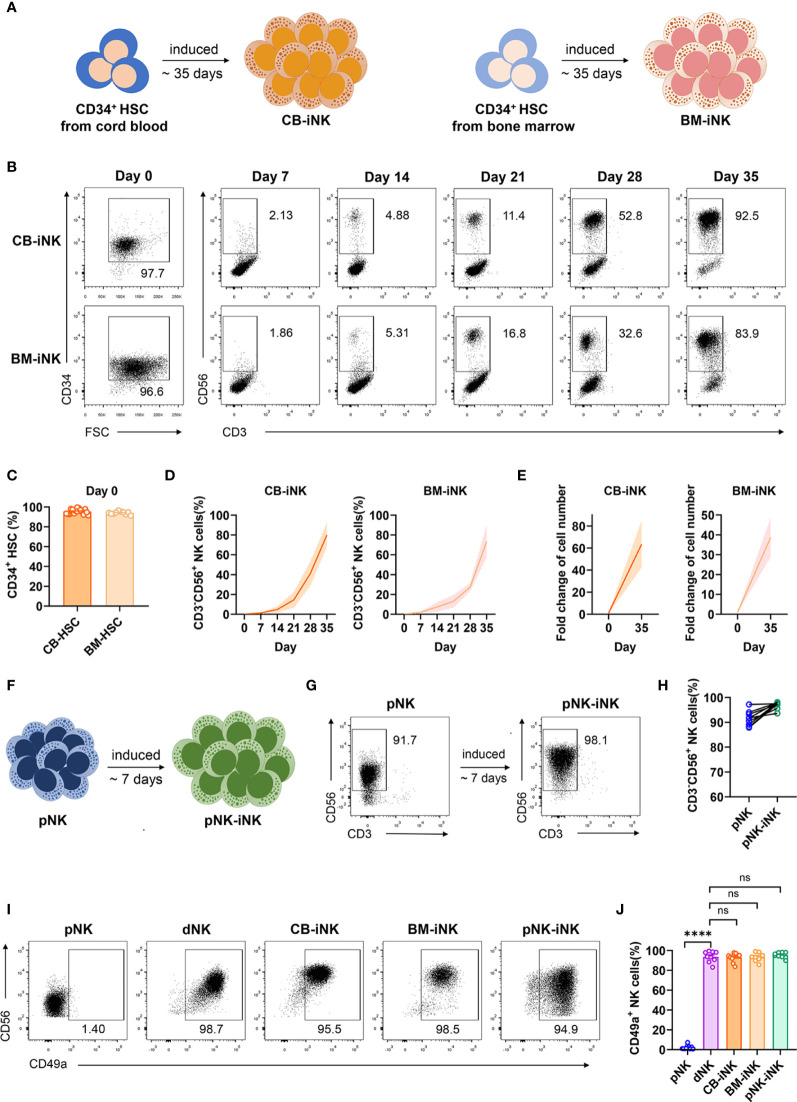
Inducing human CD49a^+^ NK cells from three different sources. **(A)** Schematic diagram of CB-iNK cells (left) and BM-iNK cells (right) acquired from HSCs. **(B)** Representative density plots of CD34^+^ cells sorted from cord blood or bone marrow mononuclear cells, and flow cytometric analysis of the percentage of CD3^-^CD56^+^ NK cells in the system on days seven through thirty-five. **(C)** Statistical analysis of CD34^+^ HSCs purity after MACS sorting. **(D, E)** Flow cytometry analysis of frequency **(D)** and absolute number **(E)** for CD3^-^CD56^+^ NK cells on day 35 of culture. **(F)** Schematic diagram of pNK-iNK cells acquired from purified pNK cells. **(G)** Representative density plots of CD3^-^CD56^+^ pNK cells and pNK-iNK cells. **(H)** Statistical analysis of CD3^-^CD56^+^ NK cells. **(I, J)** The percentage of CD3^-^CD56^+^ NK cells that expressed CD49a in each group was tested by flow cytometry. Representative density plots **(I)** and statistical calculation of all samples **(J)**. Data was analyzed by one-way ANOVA. Data represent means ± SD, n ≥ 6 in each group. ****p < 0.0001; ns, not significant.

We previously established an *in vitro* cytokine-based feeder-free system in which bone marrow progenitor cells of mice differentiate into CD49a^+^ uterine trNK cells (30). To investigate whether CD49a^+^ uterine trNK cells can be induced from human CD34^+^ HSCs, we isolated CD34^+^ HSCs from human umbilical cord blood (CB) or bone marrow (BM), and induced their differentiation into NK cells using multiple cytokine cocktails without feeders **(****Figure 1A****)**. The purity of HSCs was more than 90% confirmed using flow cytometric analysis after magnetic bead sorting **(****Figure 1B left, C****)**. The cells were cultured and induced in stages using different cytokine combinations, including Flt3L, SCF, and IL-15. The use of Flt3L and SCF promoted HSCs proliferation and transition to CLPs in the first stage. At the end of day 14, the dosage of Flt3L and SCF gradually decreased until it was no longer added. IL-15 was added on day 4 of culturing to direct CLPs toward NK cells. Gradual increases in the proportion of NK cells were observed in the culture system over time. By 35 days, over 80% of the cells were CD3^-^CD56^+^ NK cells **(****Figure 1B right, D****)**, which increased the number of cells in the culture system by 40- to 60-fold **(****Figure 1E****)**. We named the induced NK (iNK) cells derived from cord blood HSCs as CB-iNK cells and from bone marrow HSCs as BM-iNK cells.

Human chorionic gonadotropin (hCG) levels can be detected on the first day of implantation, and peaked around gestational week 12, after which they diminished during the remainder of the pregnancy (31). The phase of existence is similar with dNKs at maternal-fetal interface. And hCG can reduce the activation of cytotoxic NK cells and coordinate the vascularization of the placental bed (32). To investigate whether the combination of hCG, hypoxia, and TGF-β1 could functionally transform pNK cells into CD49a^+^ NK cells, we purified pNK cells *via* magnetic bead sorting and induced them under the compound conditions to obtain iNK cells **(****Figure 1F****)**. We named the iNK cells derived from pNK cells as pNK-iNK cells. After approximately seven days of induction, the proportion of NK cells in the culture system still exceeded 90% and flow cytometry results demonstrated that the cells had a higher expression of CD56 **(****Figures 1G, H****)**.

Next, the percentage of iNK cells that expressed CD49a in each group was tested *via* flow cytometry. The results showed that CD49a-positive cells accounted for over 90% of all NK cells in iNK group and dNK group, and less than 5% in pNK group **(****Figures 1I, J****)**. As such, we successfully established three feeder-free systems to induce CD49a^+^ NK cells *in vitro*.

### The iNK Cells With Proliferation Potential Exhibit Phenotype Signatures Similar to dNK Cells

**Figure 2:**
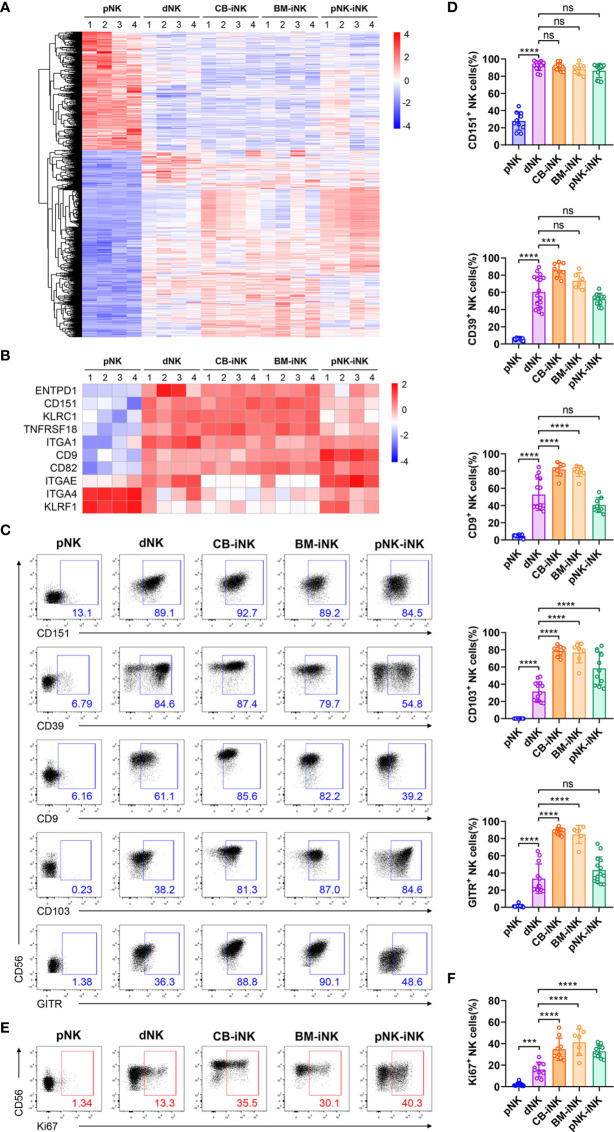
Phenotypic and proliferation potential analyses of iNK cells. **(A)** The 1116 common differentially expressed genes (DEGs) (fold change > 2 and pvalue < 0.05) of pNK cells compared with other NK cells were selected for heat map analysis. Each column represents one sample (n = 4 per group). **(B)** Some genes encoding phenotypic markers of dNK cells were selected for heat map analysis. Each column represents one sample (n = 4 per group). **(C, D)** The percentage of NK cells that expressed dNK-phenotypic markers and inhibitory receptors in each group was obtained *via* flow cytometry. Representative density plots **(C)** and statistical calculation of all samples **(D)**. **(E, F)** The percentage of NK cells that expressed Ki67 in each group was performed by flow cytometry. Representative density plots **(E)** and statistical calculation of all samples **(F)**. Data were analyzed by one-way ANOVA. Data represent means ± SD, n ≥ 6 in each group. ***p < 0.005; ****p < 0.0001; ns, not significant.

To compare the differences in gene expression between the three kinds of iNK cells with freshly isolated dNK cells and pNK cells, the transcriptomes of five types of purified NK cells were profiled by RNA sequencing (RNA-Seq), including pNK, dNK, CB-iNK, BM-iNK, and pNK-iNK. We identified the differentially expressed genes (DEGs) of the other four group NK cells compared with pNK (cutoff: fold change > 2 and p-value < 0.05) **(****Supplementary Figures 1A–D****)**. Thousands of DEGs were selected **(****Supplementary Figure 1E****)**. The heat map was drawn according to the FPKM values of the common DEGs of the other four group NK cells compared to pNK **(****Supplementary Figure 1F** and **Figure 2A****)**. Our results demonstrated the iNKs were similar to the dNKs but were different from the pNKs.

Consistent with previous reports that dNKs show phenotypic features distinct from that of pNKs (33), we found three kinds of iNKs that are similar to dNKs with higher expression of specific genes than pNKs, such as encoding surface molecules (ITGA1, CD151, CD9, and ENTPD1) and inhibitory receptors (TNFRSF18 and ITGAE) **(****Figure 2B****)**. These genes are included in the genes displayed in **Figure 2A**. As a member of the tetraspanin family, CD151 and CD9 are associated with regulating cell migration and invasion and are exclusively expressed in dNKs (33). Previous works also have shown that IL-15 and TGF-β upregulated the expression of vital integrins, like CD9 and CD103 (gene name: ITGAE) (34). Besides, CD49a (gene name: ITGA1) and CD39 (gene name: ENTPD1) have previously been shown to be expressed by trNK cells in murine and human liver (18, 35, 36). Recent studies suggest that IL-15 can upregulate uNK cell expression of CD39 *in vitro*, and CD39^+^ uNK cells are more differentiated subsets (37). Flow cytometric analysis confirmed that the protein expression levels of the markers mentioned above are consistent with the results of RNA-seq: these protein expression levels were significantly higher in the dNKs and iNKs than in the pNKs **(****Figures 2C, D****)**.

The proportion of Ki67^+^ among CD3^-^CD56^+^ NK cells was quantified **(****Figures 2E, F****)**, and we found that dNKs and iNKs had more than 15% of Ki67^+^ cells, confirming their proliferation potential, while the pNKs have significantly lower proliferation potential. In addition, the iNKs also have higher proliferation potential than the dNKs.

In summary, the results of transcriptome sequencing and multicolor flow cytometric analysis demonstrated that iNK cells with proliferation potential and dNK cells exhibit similar phenotypes.

### The iNK Cells Have Low Cytotoxic Capability

**Figure 3:**
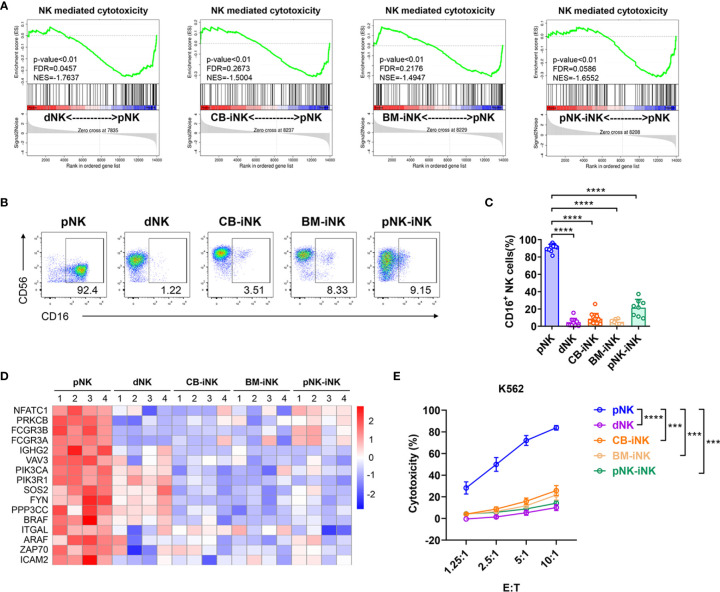
Cytotoxic capability detection of iNK cells. **(A)** Gene set enrichment analysis (GSEA) revealed an increase in Natural Killer cell-mediated cytotoxicity (enrichment plot: NATURAL KILLER CELL MEDIATED CYTOTOXICITY, HSA04650) in pNK cells compared with dNK cells and three kinds of iNK cells (n = 4 per group). **(B, C)** The percentage of CD16^+^ NK cells in each group was tested by flow cytometry. Representative density plots **(B)** and statistical calculation of all samples **(C)**. Data represent means ± SD. **(D)** Some of the NK cells mediated cytotoxicity-related genes were selected for heat map analysis (n = 4 per group). **(E)** The direct cytotoxicity of different NK cells toward K562, as measured by flow cytometry. Results are presented as the mean ± SEM. Data were analyzed by one-way ANOVA, and n ≥ 6 in each group **(C, E)**. ***p < 0.005; ****p < 0.0001.

Decidual NK cells exhibit lower cytolytic function than pNK cells, which is critical for maintaining immune tolerance at the fetal-maternal interface (8, 38). To assess the cytotoxicity of iNKs, we first performed a gene set enrichment analysis (GSEA) of the five kinds of NKs transcriptional profiles. Compared to dNK cells and iNK cells, pNK cells were significantly enriched in the pathway of natural killer-mediated cytotoxicity (KO04650) **(****Figure 3A****)**. NK cells mediate ADCC *via* its FcγRIIIA receptor, CD16 (39), which is an activation marker indicating killing function (40). The CD16^+^ NK-cell subset accounts for > 90% of pNK cells, which was significantly higher compared with < 10% of dNK cells, CB-iNK cells, and BM-iNK cells, as well as < 50% of pNK-iNK cells **(****Figures 3B, C****)**. The expression level of some NK cell-mediated cytotoxicity-associated genes is shown in the heatmap **(****Figure 3D****)**. We observed lower expression levels in dNKs and iNKs than in pNKs.

To further verify the cytotoxic activity of NK cells, we co-cultured purified NK cells (as effector cells) with myeloid leukemia-derived cell lines K562 (as target cell), which is NK cell-susceptible. After five hours, the dead K562 cells (7AAD^+^) were analyzed by flow cytometric at different effect-target ratios. pNK cells were significant efficient at killing K562 than dNK cells and other iNK cells **(****Figure 3E****)**. Overall, iNK cells have much lower cytotoxic capability than pNK cells, which is similar to dNK cells.

### The iNK Cells Present the Functional Characterization of dNK Cells

**Figure 4:**
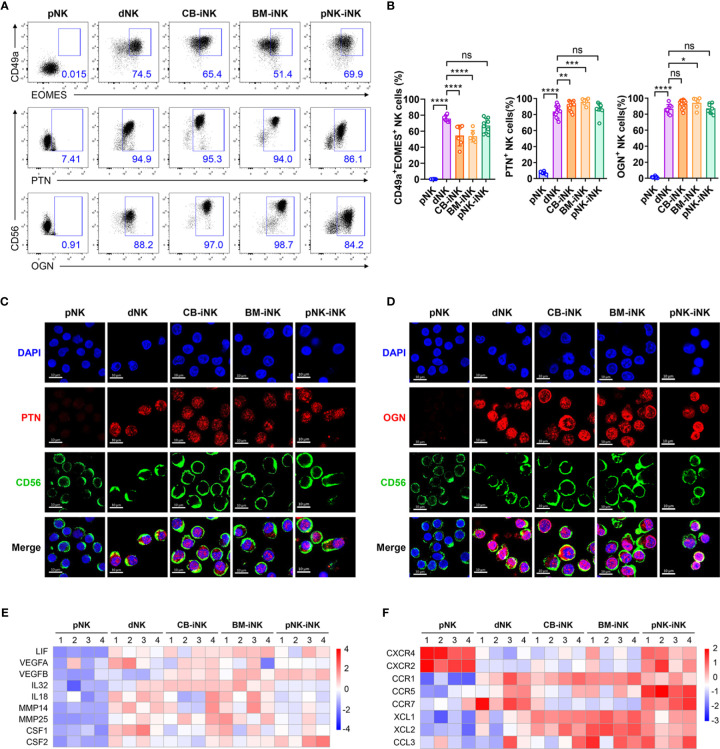
The iNK cells have high expression of GPFs, proangiogenic factors, chemokines and chemokine receptors like dNK cells. **(A, B)** The percentage of CD49a^+^EOMES^+^ and GFP^+^ NK cells in each group was tested by flow cytometry. Representative density plots **(A)** and statistical calculation of all samples **(B)**. Data represent means ± SD, n ≥ 6 in each group. Data were analyzed by one-way ANOVA. *p < 0.05; **p < 0.01; ***p < 0.005; ****p < 0.0001; ns, not significant. **(C, D)** CLSM images showing the secretion of PTN **(C)** and OGN **(D)** in pNKs, dNKs and other iNKs. Scale bars, 10 μm. **(E)** Some genes encoding the proangiogenic factors were selected for heat map analysis. **(F)** Some genes encoding the chemokines and chemokine-receptors were selected for heat map analysis.

Our group previously found that decidual CD49a^+^ NK cells with high expression of EOMES secrete several growth-promoting factors (GPFs), including pleiotrophin (PTN) and osteoglycin (OGN) (13). Given that almost all iNK cells are CD49a-positive **(****Figure 1I****)**, we next investigated whether iNK cells express EOMES and secrete GPFs. This result showed that the three kinds of iNK cells had a high proportion of CD49a^+^EOMES^+^ cells similar to dNK cells. Besides, the PTN^+^ NK-cell subset accounts for > 90% of CB-iNK cells and BM-iNK cells, as well as > 60% of pNK-iNK cells. Similarly, the OGN^+^ NK-cell subset accounts for > 90% of CB-iNK cells and BM-iNK cells, as well as > 80% of pNK-iNK cells **(****Figures 4A, B****)**. A similar result was observed using confocal laser scanning microscopy (CLSM). PTN and OGN were marked in red and CD56 was marked in green **(****Figures 4C, D****)**.

We also found that several genes related to blood vessels and embryonic development in iNK cells were transcribed at a high level, similar to dNK cells. Producing vascular endothelial growth factor (VEGF), dNKs promote blood vessel growth and help reshape the spiral arteries to increase the supply of blood to the implantation site (41–43). Leukemia inhibitory factor (LIF) is associated with STAT3 activation, is produced by dNKs at the fetal-maternal interface, and can regulate trophoblast invasion (44, 45). Activated KIR2DS1^+^ dNKs can produce granulocyte-macrophage colony-stimulating factor (GM-CSF), which enhanced the migration of primary trophoblasts in favor of placentation (46). The heatmap displays that expression levels of these genes were higher in dNK cells and iNK cells than in pNK cells, including VEGFA, LIF, IL-32, and CSF2 **(****Figure 4E****)**.

Chemokines and their receptors also play a critical role in pregnancy. CXCR4 was the chemokine receptor for the ligands CXCL12, which were secreted by extravillous trophoblasts (EVT) (47). The pNK-iNK cells expressed CXCR4, which mediate the migration of uNKs into early pregnancy decidua (48) **(****Figure 4F****)**. This indicates that pNK-iNK cells could migrate functionally from the periphery to the uterus. Other chemokines and chemokine receptors, like CCR5, CCR7, XCL1, and CCL3, all played a beneficial role during pregnancy and were highly expressed in dNK cells and iNK cells **(****Figure 4F****)**.

Overall, iNK cells are functionally similar to dNK cells by secreting several GPFs and highly expressed genes encoding proangiogenic factors, chemokines, and chemokine receptors.

### The iNK Cells Promote Fetal Growth in Mice

To assess whether human iNK cells have a functional distinguished signature and can nourish the uterine microenvironment, we first analyzed the amino acid sequence homology among some functional molecules of dNKs between humans and mice. The data demonstrated that several key proteins were highly conserved, including PTN, OGN, VEGF, and MMPs. The cytokines, chemokines, and phenotypic molecules of humans and mice share considerable amino acid sequence identities **(****Supplementary Figure 2****)**.

**Figure 5:**
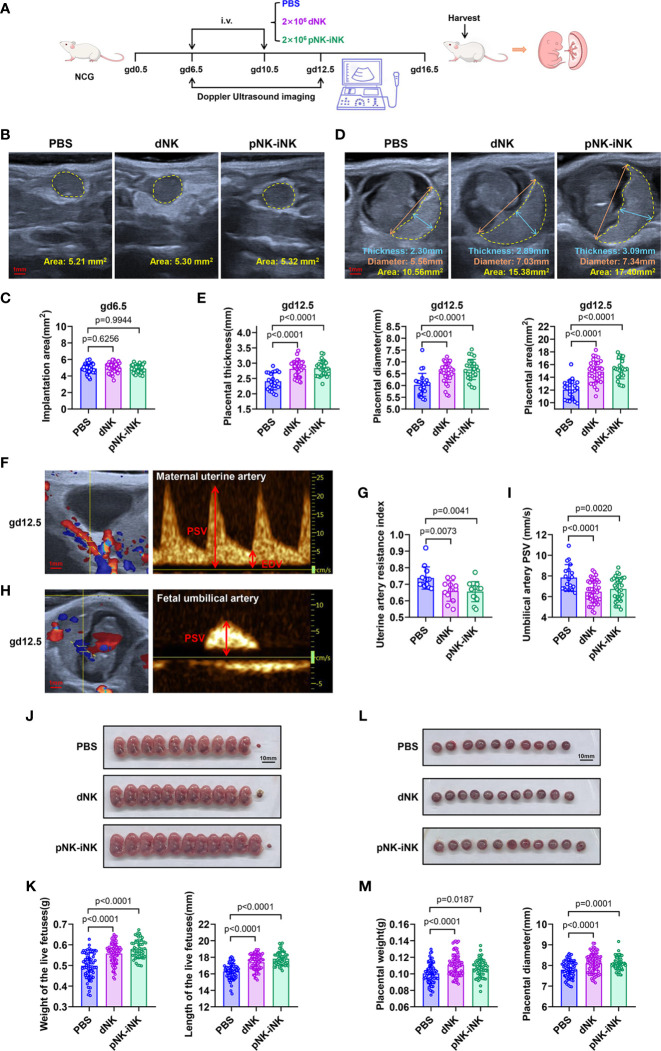
Adoptive transferred pNK-iNK cells promote fetal growth. **(A)** Schematic diagram of adoptive transferring pNK-iNK cells in pregnancy mice model, n=6 pregnancy mice per group. The experiment was performed twice and the results were shown only once. **(B, C)** Implantation areas of pregnancy mice before being transferred cells at gd6.5. Bar, 1 mm. Representative ultrasound images from the three groups **(B)** and statistical calculations **(C)** are shown. Results are presented as individual values for each implantation and mean. **(D, E)** Placental measurements at gd12.5. Bar, 1 mm. **(D)** Representative ultrasound image of the single fetus, showing placental thickness, placental diameter, and placental area. **(E)** Statistical calculation of the measurements is shown. Results are presented as individual values for each placenta and mean. **(F–I)** Analysis of blood flow velocities at gd12.5. Bar, 1 mm. Representative CF (left) and PW (right) doppler images showing PSV and EDV of UA **(F)** and UmA **(H)**. **(G)** Statistical calculation of UA resistance index (2 experimental repeats were combined). **(I)** Statistical calculation of UmA peak systolic velocities. **(J–M)** The pregnancy outcome of adoptive transferring NK cells in mice. Bar, 10 mm. **(J)** Representative pictures of fetuses from NK-cells transferred mice and control mice (PBS transferred) at gd16.5. **(K)** Statistics were calculated by the weight and length of the live fetus in different groups. **(L)** Representative pictures of the placenta correspond to the fetus in **(J)**. **(M)** Statistics were calculated by the weight and diameter of the placenta. Data represent means ± SD. Data were analyzed by one-way ANOVA **(C, E, G, I, K, M)**.

Second, to test the biological function of iNK cells *in vivo*, we performed adoptive transfer experiments in a mouse pregnancy model **(****Figure 5A****)**. After we documented the implantations *via* doppler ultrasound imaging at gd6.5, the NCG mice were randomly divided into three groups, with no significant difference in the implantation area of the single embryo **(****Figures 5B, C****)**. In mice, dNK cells on day 6.5 of gestation, proliferated, and peaked on day 10.5-12.5 of gestation. They then declined from mid-gestation onwards (49). Therefore, mice in the PBS group, the dNK group, and the pNK-iNK group received a transfusion of PBS, dNK cells (2×10^6^ cell/mouse), and pNK-iNK cells (2×10^6^ cell/mouse) at gd6.5 and gd10.5, respectively. We then performed ultrasound measurements of placental thickness, diameter, and area at gd12.5 **(****Figure 5D****)**. By this time, the size of the placenta can be detected and is not too large for the probe. The placental thicknesses, diameters, and areas in the dNK group and pNK-iNK group were significantly (p<0.0001) higher than in the PBS group **(****Figure 5E****)**. Our results indicate that transferred dNK cells and pNK-iNK cells dilated placental dimensions.

Additionally, the blood velocity parameters of the maternal uterine artery (UA) and fetal umbilical artery (UmA) were evaluated by doppler measurements at gd12.5. The UA is located behind the bladder **(****Figure 5F**, left), while the UmA is located between the fetus and placenta **(****Figure 5H**, left). We measured the peak systolic velocities (PSVs) and end-diastolic velocities (EDVs) to calculate the resistance index (RI) **(****Figures 5F, H**, right). The EDV of UmA is absent at this gestational age. The RI of UA **(****Figure 5G****)** and the PSV of UmA **(****Figure 5I****)** in the dNK group and the pNK-iNK group decreased compared to the PBS group, which was an indication of better vascularization with more conducive to fetal well-being.

The mice were euthanized at gd16.5 and we found that the weight and length of the live fetuses injected with dNK cells or pNK-iNK cells were significantly higher compared to the PBS-transferred controls **(****Figures 5J, K****)**. The weight and diameter of the placenta, corresponding to each live fetus, were simultaneously measured. These placentas had greater weight and a larger diameter in the dNK group and the pNK-iNK group **(****Figures 5L, M****)**. We also performed a similar experiment with adoptive transferring CB-iNK cells **(****Supplementary Figure 3A****)**. The dNK group and the CB-iNK group had live fetuses with greater weight and length compared to the PBS group **(****Supplementary Figures 3B, C****)**. In addition, the pregnant recipient mice were euthanized, and the mononuclear cells of the uterus, blood, bone marrow, liver, spleen and lung were examined by flow cytometry using anti-human antibodies at 24 h post cell transfer. The results showed that human NK cells were detected in the uterus and lung of recipient mice, which indicated that the injected NK cells homed to uterus *via* the tail vein (lung metastatic models). And the NK cells homing to uterus were still CD49a positive **(****Supplementary Figure 3D****)**.

Altogether, these results confirm that iNK cells can promote fetal growth and improve uterine artery blood flow.

## Discussion

In this study, we developed three platforms that support *in vitro* generation of human CD49a^+^ NK cells, which are distinguished by a decidual-like signature and can promote fetal growth *in vivo*. Previous *in vitro* investigations focused on the analysis of induced NK cells *via* the single resource through a few markers or cytokines, which are insufficient to assign lineage identity (22). Considering clinical ethics requirements and the situation of the patients, we studied and compared three different induction platforms from umbilical cord blood HSCs, bone marrow HSCs, and pNKs. After 35 days of long-term differentiation from HSCs or seven days of short-term polarization from pNKs, we found that all iNK cells from the three induction platforms yielded cells that were phenotypically and functionally similar to dNK cells. Specifically, the BM-iNK cells and pNK-iNK cells can be induced from autologous bone marrow HSCs and pNKs, respectively, which meet the requirements of medical ethics for autologous cell therapy. Compared to 35 days of long-term differentiation of BM-iNK cells and CB-iNK cells, the pNK-iNK cells were induced over a relatively short period. Previous studies presented several systems for the generation of NK cells or other innate lymphoid cells from HSCs in the presence of feeder cells, which inevitably increases the risk of introducing exogenous pollutants (50). Our iNK cells from three different platforms were cultured without feeder cells and are sufficient to meet various clinical requirements. Supplementing a certain amount of functional exogenous dNK cells to uterus may become a potential therapeutic modality for patients with pregnancy disorder. While, there is currently no way available to obtain dNK cells in clinical use. It is possible that our induced systems may compensate the situation. And we are now taking forward the research of clinical applications of iNK cells.

The origin of uNKs has long been controversial. Data exist in support of both sides, as uNK cell precursors have been locally identified in the endometrium (51) and pNK cells can acquire an uNK cell phenotype upon TGF-β1 or Hypoxia exposure (25–27, 52). Studies assessing human uterus transplantation and monozygotic twins studies provide evidence that uNKs can be replenished *via* circulation (37). The successful induction of CD49a^+^ tissue-resident-like NK cells in our study further demonstrates the plasticity of NK cells. Our results from the RNA-seq analysis comparison further confirm this finding, where the GO terms “angiogenesis”, “decidua related GPFs”, “chemokines and chemokine receptors,” not “cytotoxicity,” were enriched in iNK cells from the three platforms. Our data indicate that uNKs can be transiently tissue-resident-like cells from both pNK and HSC precursor cells based on their expressions of tissue residency markers. Therefore, the decidual microenvironment is critical to induce or maintain both the phenotype and function of dNKs. However, the exact mechanisms for the differentiation of decidual-like NK cells from pNKs or HSCs must be examined in future studies that assess both healthy women and the pathological conditions present in pregnancy disorders.

*Ex vivo*-induced CD49a^+^ iNK cells can mimic dNK cell function *in vivo*. Several attempts have been made to expand NK cells with phenotypic similarities to dNK cells (27, 53). However, it is unclear whether these NK cells induced by different pathways have *in vivo* functions that promote embryonic development. In this study, we used doppler ultrasound imaging to observe the mouse and fetal states in pregnant mice with no pathological damage, providing significant insight into pregnancy in an animal model. Based on our data analysis and results, we found that iNK cells reduced uterine artery resistance and promote the growth and development of the fetus *in vivo*.

In summary, our data demonstrates that the CD49a^+^ iNK cells with phenotypes and functions similar to dNK cells can promote the growth and development of the fetus. Therefore, supplementing CD49a^+^ iNK cells to the uterus could have significant value in treating reproductive disorders associated with NK cells, including intrauterine growth restriction and repeated miscarriages.

## Data Availability Statement

The datasets presented in this study can be found in online repositories. The names of the repository/repositories and accession number(s) can be found below: https://www.ncbi.nlm.nih.gov/, GSE184719.

## Ethics Statement

The studies involving human participants were reviewed and approved by The Ethics Committee of the University of Science and Technology of China. The patients/participants provided their written informed consent to participate in this study. The animal study was reviewed and approved by The Ethics Committee of the University of Science and Technology of China.

## Author Contributions

BF, HW, and XD conceived and conducted the project. BF, HW, and HZ supervised the project. XD performed the experiments, analyzed and interpreted data, and wrote the manuscript. DJ and ZN contributed to adoptive transfer experiment. JZ, YZ, and XZ contributed to some imaging analysis and data interpretation. HZ and XT contributed to collect samples and information from donors. All authors contributed to the article and approved the submitted version.

## Funding

This work was supported by the National Key Research & Developmental Program of China (2018YFC1003900 to HW), the National Natural Science Foundation of China (81930037 to HW; 31870914, 81922028 to BF), Youth Innovation Promotion Association of Chinese Academy of Sciences (Grant 2019442 to BF), the Strategic Priority Research Program of the Chinese Academy of Sciences (XDB39020700 to BF).

## Conflict of Interest

The authors declare that the research was conducted in the absence of any commercial or financial relationships that could be construed as a potential conflict of interest.

## Publisher’s Note

All claims expressed in this article are solely those of the authors and do not necessarily represent those of their affiliated organizations, or those of the publisher, the editors and the reviewers. Any product that may be evaluated in this article, or claim that may be made by its manufacturer, is not guaranteed or endorsed by the publisher.

## References

[1] Red-Horse K Zhou Y Genbacev O Prakobphol A Foulk R McMaster M. Trophoblast Differentiation During Embryo Implantation and Formation of the Maternal-Fetal Interface. J Clin Invest (2004) 114:744–54. doi: 10.1172/JCI200422991 PMC516273 15372095

[2] Trundley A Moffett A. Human Uterine Leukocytes and Pregnancy. Tissue Antigens (2004) 63:1–12. doi: 10.1111/j.1399-0039.2004.00170.x 14651517

[3] Gomez-Lopez N Guilbert LJ Olson DM. Invasion of the Leukocytes Into the Fetal-Maternal Interface During Pregnancy. J Leukocyte Biol (2010) 88:625–33. doi: 10.1189/jlb.1209796 20519637

[4] Moffett-King A. Natural Killer Cells and Pregnancy. Nat Rev Immunol (2002) 2:656–63. doi: 10.1038/nri886 12209134

[5] Pegram HJ Andrews DM Smyth MJ Darcy PK Kershaw MH. Activating and Inhibitory Receptors of Natural Killer Cells. Immunol Cell Biol (2011) 89:216–24. doi: 10.1038/icb.2010.78 20567250

[6] Kopcow HD Allan DSJ Chen X Rybalov B Andzelm MM Ge B. Human Decidual NK Cells Form Immature Activating Synapses and Are Not Cytotoxic. Proc Natl Acad Sci USA (2005) 102:15563–8. doi: 10.1073/pnas.0507835102 PMC1266146 16230631

[7] Sivori S Vacca P Del Zotto G Munari E Mingari MC Moretta L. Human NK Cells: Surface Receptors, Inhibitory Checkpoints, and Translational Applications. Cell Mol Immunol (2019) 16:430–41. doi: 10.1038/s41423-019-0206-4 PMC6474200 30778167

[8] Fu B Li X Sun R Tong X Ling B Tian Z. Natural Killer Cells Promote Immune Tolerance by Regulating Inflammatory TH17 Cells at the Human Maternal–Fetal Interface. Proc Natl Acad Sci USA (2013) 110:E231–40. doi: 10.1073/pnas.1206322110 PMC3549088 23271808

[9] Croy BA He H Esadeg S Wei Q McCartney D Zhang J. Uterine Natural Killer Cells: Insights to Their Cellular and Molecular Biology From Mouse Modelling. Reproduction (2003) 126:149–60. doi: 10.1530/rep.0.1260149 PMC2967520 12887272

[10] Zhang J Chen Z Smith GN Croy BA. Natural Killer Cell-Triggered Vascular Transformation: Maternal Care Before Birth? Cell Mol Immunol (2011) 8:1–11. doi: 10.1038/cmi.2010.38 20711229 PMC3079746

[11] Ashkar AA Di Santo JP Croy BA. Interferon Gamma Contributes to Initiation of Uterine Vascular Modification, Decidual Integrity, and Uterine Natural Killer Cell Maturation During Normal Murine Pregnancy. J Exp Med (2000) 192:259–70. doi: 10.1084/jem.192.2.259 PMC2193246 10899912

[12] De Oliveira LG Lash GE Murray-Dunning C Bulmer JN Innes BA Searle RF. Role of Interleukin 8 in Uterine Natural Killer Cell Regulation of Extravillous Trophoblast Cell Invasion. Placenta (2010) 31:595–601. doi: 10.1016/j.placenta.2010.04.012 20483454

[13] Fu B Zhou Y Ni X Tong X Xu X Dong Z. Natural Killer Cells Promote Fetal Development Through the Secretion of Growth-Promoting Factors. Immunity (2017) 47:1100–1113.e6. doi: 10.1016/j.immuni.2017.11.018 29262349

[14] Robertson SA Roberts CT Farr KL Dunn AR Seamark RF. Fertility Impairment in Granulocyte-Macrophage Colony-Stimulating Factor-Deficient Mice1. Biol Reprod (1999) 60:251–61. doi: 10.1095/biolreprod60.2.251 9915988

[15] Fu B Tian Z Wei H. TH17 Cells in Human Recurrent Pregnancy Loss and Pre-Eclampsia. Cell Mol Immunol (2014) 11:564–70. doi: 10.1038/cmi.2014.54 PMC4220838 25027967

[16] Yougbaré I Tai W-S Zdravic D Oswald BE Lang S Zhu G. Activated NK Cells Cause Placental Dysfunction and Miscarriages in Fetal Alloimmune Thrombocytopenia. Nat Commun (2017) 8:224. doi: 10.1038/s41467-017-00269-1 28794456 PMC5550461

[17] Zhou Y Fu B Xu X Zhang J Tong X Wang Y. PBX1 Expression in Uterine Natural Killer Cells Drives Fetal Growth. Sci Transl Med (2020) 12:eaax1798. doi: 10.1126/scitranslmed.aax1798 32238574

[18] Peng H Jiang X Chen Y Sojka DK Wei H Gao X. Liver-Resident NK Cells Confer Adaptive Immunity in Skin-Contact Inflammation. J Clin Invest (2013) 123:1444–56. doi: 10.1172/JCI66381 PMC3613925 23524967

[19] Sojka DK Plougastel-Douglas B Yang L Pak-Wittel MA Artyomov MN Ivanova Y. Tissue-Resident Natural Killer (NK) Cells are Cell Lineages Distinct From Thymic and Conventional Splenic NK Cells. eLife (2014) 3:e01659. doi: 10.7554/eLife.01659 24714492 PMC3975579

[20] Tong X Gao M Du X Lu F Wu L Wei H. Analysis of Uterine CD49a+ NK Cell Subsets in Menstrual Blood Reflects Endometrial Status and Association With Recurrent Spontaneous Abortion. Cell Mol Immunol (2021) 18:1838–40. doi: 10.1038/s41423-021-00687-8 PMC8245401 34002045

[21] Di Santo JP. Natural Killer Cell Developmental Pathways: A Question of Balance. Annu Rev Immunol (2006) 24:257–86. doi: 10.1146/annurev.immunol.24.021605.090700 16551250

[22] Renoux VM Zriwil A Peitzsch C Michaëlsson J Friberg D Soneji S. Identification of a Human Natural Killer Cell Lineage-Restricted Progenitor in Fetal and Adult Tissues. Immunity (2015) 43:394–407. doi: 10.1016/j.immuni.2015.07.011 26287684

[23] Colucci F Di Santo JP. The Receptor Tyrosine Kinase C-Kit Provides a Critical Signal for Survival, Expansion, and Maturation of Mouse Natural Killer Cells. Blood (2000) 95:984–91. doi: 10.1182/blood.V95.3.984.003k40_984_991 10648413

[24] Freud AG Becknell B Roychowdhury S Mao HC Ferketich AK Nuovo GJ. A Human CD34(+) Subset Resides in Lymph Nodes and Differentiates Into CD56brightNatural Killer Cells. Immunity (2005) 22:295–304. doi: 10.1016/j.immuni.2005.01.013 15780987

[25] Keskin DB Allan DSJ Rybalov B Andzelm MM Stern JNH Kopcow HD. Tgfβ Promotes Conversion of CD16+ Peripheral Blood NK Cells Into CD16– NK Cells With Similarities to Decidual NK Cells. Proc Natl Acad Sci USA (2007) 104:3378–83. doi: 10.1073/pnas.0611098104 PMC1805591 17360654

[26] Parodi M Raggi F Cangelosi D Manzini C Balsamo M Blengio F. Hypoxia Modifies the Transcriptome of Human NK Cells, Modulates Their Immunoregulatory Profile, and Influences NK Cell Subset Migration. Front Immunol (2018) 9:2358. doi: 10.3389/fimmu.2018.02358 30459756 PMC6232835

[27] Cerdeira AS Rajakumar A Royle CM Lo A Husain Z Thadhani RI. Conversion of Peripheral Blood NK Cells to a Decidual NK-Like Phenotype by a Cocktail of Defined Factors. J Immunol (2013) 190:3939–48. doi: 10.4049/jimmunol.1202582 PMC3742368 23487420

[28] Cavalli RC Cerdeira AS Pernicone E Korkes HA Burke SD Rajakumar A. Induced Human Decidual NK-Like Cells Improve Utero-Placental Perfusion in Mice. PloS One (2016) 11:e0164353. doi: 10.1371/journal.pone.0164353 27736914 PMC5063315

[29] Jedema I van der Werff NM Barge RMY Willemze R Falkenburg JHF. New CFSE-Based Assay to Determine Susceptibility to Lysis by Cytotoxic T Cells of Leukemic Precursor Cells Within a Heterogeneous Target Cell Population. Blood (2004) 103:2677–82. doi: 10.1182/blood-2003-06-2070 14630824

[30] Ni X Fu B Zhang J Sun R Tian Z Wei H. Cytokine-Based Generation of CD49a+Eomes–/+ Natural Killer Cell Subsets. Front Immunol (2018) 0:2126. doi: 10.3389/fimmu.2018.02126 PMC6167425 30319610

[31] Cole LA. New Discoveries on the Biology and Detection of Human Chorionic Gonadotropin. Reprod Biol Endocrinol (2009) 7:8. doi: 10.1186/1477-7827-7-8 19171054 PMC2649930

[32] Bansal AS Bora SA Saso S Smith JR Johnson MR Thum M-Y. Mechanism of Human Chorionic Gonadotrophin-Mediated Immunomodulation in Pregnancy. Expert Rev Clin Immunol (2012) 8:747–53. doi: 10.1586/eci.12.77 23167686

[33] Koopman LA Kopcow HD Rybalov B Boyson JE Orange JS Schatz F. Human Decidual Natural Killer Cells Are a Unique NK Cell Subset With Immunomodulatory Potential. J Exp Med (2003) 198:1201–12. doi: 10.1084/jem.20030305 PMC2194228 14568979

[34] Hawke LG Mitchell BZ Ormiston ML. TGF-β and IL-15 Synergize Through MAPK Pathways to Drive the Conversion of Human NK Cells to an Innate Lymphoid Cell 1–Like Phenotype. JI (2020) 204:3171–81. doi: 10.4049/jimmunol.1900866 32332109

[35] Zhou J Peng H Li K Qu K Wang B Wu Y. Liver-Resident NK Cells Control Antiviral Activity of Hepatic T Cells. via PD-1-PD-L1 Axis Immun (2019) 50:403–17.e4. doi: 10.1016/j.immuni.2018.12.024 30709740

[36] Gill US Pallett LJ Thomas N Burton AR Patel AA Yona S. Fine Needle Aspirates Comprehensively Sample Intrahepatic Immunity. Gut (2019) 68:1493–503. doi: 10.1136/gutjnl-2018-317071 PMC6691856 30487267

[37] Strunz B Bister J Jönsson H Filipovic I Crona-Guterstam Y Kvedaraite E. Continuous Human Uterine NK Cell Differentiation in Response to Endometrial Regeneration and Pregnancy. Sci Immunol (2021) 6:eabb7800. doi: 10.1126/sciimmunol.abb7800 33617461

[38] Fu B Tian Z Wei H. Subsets of Human Natural Killer Cells and Their Regulatory Effects. Immunology (2014) 141:483–9. doi: 10.1111/imm.12224 PMC3956422 24303897

[39] Trotta R Ciarlariello D Dal Col J Mao H Chen L Briercheck E. The PP2A Inhibitor SET Regulates Granzyme B Expression in Human Natural Killer Cells. Blood (2011) 117:2378–84. doi: 10.1182/blood-2010-05-285130 PMC3062407 21156847

[40] Borrego F Robertson MJ Ritz J Peña J Solana R. CD69 Is a Stimulatory Receptor for Natural Killer Cell and its Cytotoxic Effect Is Blocked by CD94 Inhibitory Receptor. Immunology (1999) 97:159–65. doi: 10.1046/j.1365-2567.1999.00738.x PMC2326810 10447727

[41] Hanna J Goldman-Wohl D Hamani Y Avraham I Greenfield C Natanson-Yaron S. Decidual NK Cells Regulate Key Developmental Processes at the Human Fetal-Maternal Interface. Nat Med (2006) 12:1065–74. doi: 10.1038/nm1452 16892062

[42] Kalkunte SS Mselle TF Norris WE Wira CR Sentman CL Sharma S. VEGF C Facilitates Immune Tolerance and Endovascular Activity of Human Uterine NK Cells at the Maternal-Fetal Interface. J Immunol (2009) 182:4085–92. doi: 10.4049/jimmunol.0803769 PMC3616376 19299706

[43] Lash GE Naruse K Innes BA Robson SC Searle RF Bulmer JN. Secretion of Angiogenic Growth Factors by Villous Cytotrophoblast and Extravillous Trophoblast in Early Human Pregnancy. Placenta (2010) 31:545–8. doi: 10.1016/j.placenta.2010.02.020 20338637

[44] Poehlmann TG Fitzgerald JS Meissner A Wengenmayer T Schleussner E Friedrich K. Trophoblast Invasion: Tuning Through LIF, Signalling *via* Stat3. Placenta (2005) 26:S37–41. doi: 10.1016/j.placenta.2005.01.007 15837065

[45] Reister F Kingdom JCP Ruck P Marzusch K Heyl W Pauer U. Altered Protease Expression by Periarterial Trophoblast Cells in Severe Early-Onset Preeclampsia With IUGR. J Perinatal Med (2006) 34:272–9. doi: 10.1515/JPM.2006.052 16856814

[46] Xiong S Sharkey AM Kennedy PR Gardner L Farrell LE Chazara O. Maternal Uterine NK Cell–Activating Receptor KIR2DS1 Enhances Placentation. J Clin Invest (2013) 123:4264–72. doi: 10.1172/JCI68991 PMC4382274 24091323

[47] Hanna J Wald O Goldman-Wohl D Prus D Markel G Gazit R. CXCL12 Expression by Invasive Trophoblasts Induces the Specific Migration of CD16– Human Natural Killer Cells. Blood (2003) 102:1569–77. doi: 10.1182/blood-2003-02-0517 12730110

[48] Hannan NJ Jones RL White CA Salamonsen LA. The Chemokines, CX3CL1, CCL14, and CCL4, Promote Human Trophoblast Migration at the Feto-Maternal Interface1. Biol Reprod (2006) 74:896–904. doi: 10.1095/biolreprod.105.045518 16452465

[49] Sojka DK Yang L Yokoyama WM. Uterine Natural Killer Cells: To Protect and to Nurture. Birth Defects Res (2018) 110:1531–8. doi: 10.1002/bdr2.1419 PMC6309490 30467993

[50] Hernández DC Juelke K Müller NC Durek P Ugursu B Mashreghi M-F. An In Vitro Platform Supports Generation of Human Innate Lymphoid Cells From CD34+ Hematopoietic Progenitors That Recapitulate Ex Vivo Identity. Immunity (2021) 54:2417–32. doi: 10.1016/j.immuni.2021.07.019 34453879

[51] Vacca P Vitale C Montaldo E Conte R Cantoni C Fulcheri E. CD34+ Hematopoietic Precursors are Present in Human Decidua and Differentiate Into Natural Killer Cells Upon Interaction With Stromal Cells. Proc Natl Acad Sci (2011) 108:2402–7. doi: 10.1073/pnas.1016257108 PMC3038730 21248224

[52] Carlino C Stabile H Morrone S Bulla R Soriani A Agostinis C. Recruitment of Circulating NK Cells Through Decidual Tissues: A Possible Mechanism Controlling NK Cell Accumulation in the Uterus During Early Pregnancy. Blood (2008) 111:8. doi: 10.1182/blood-2007-08-105965 18187664

[53] Euchner J Sprissler J Cathomen T Fürst D Schrezenmeier H Debatin K-M. Natural Killer Cells Generated From Human Induced Pluripotent Stem Cells Mature to CD56brightCD16+NKp80+/-*In-Vitro* and Express KIR2DL2/DL3 and KIR3DL1. Front Immunol (2021) 12:640672. doi: 10.3389/fimmu.2021.640672 34017328 PMC8129508

