# Supplementary material for: Human-Induced CD49a+ NK Cells Promote Fetal Growth
Source: Front Immunol. 2022 Feb 4;13:821542. doi: 10.3389/fimmu.2022.821542 (PMC8854499; doi:10.3389/fimmu.2022.821542)
Supplement: Supplementary file 1 [file DataSheet_1.docx]

Supplementary Material

Human-induced CD49a^+^ NK cells promote fetal growth

# Supplementary Materials and Methods

**Mice.** Immunodeficient NCG (NOD/ShiLtJGpt-Prkdc^em26Cd52^Il2rg^em26Cd22^/Gpt) mice were purchased from GemPharmatech Co., Ltd., Nanjing, China. All mice were maintained under pathogen-free conditions. All experimental procedures involving mice followed the National Guidelines for Animal Usage in Research (China) and were approved by the Ethics Committee of the University of Science and Technology of China (Reference NO. USTCACUC1801018).

**Differentially expressed genes (DEGs).** RNA differential expression analysis was performed using DESeq2 software between two different groups. The genes/transcripts with p-value<0.05 and absolute fold change>2 were considered differentially expressed genes/transcripts.

**Phylogenic homology analysis.** Protein sequences were identified using the NCBI HomoloGene system to analyze the distinguishing features between proteins in human NK cells and mice. Using protein basic local alignment search tool (BLAST) (NCBI, https://blast.ncbi.nlm.nih.gov/Blast.cgi) for the identity analysis, the results are presented as a percentage.

**Adoptive transfer of NK cells.** NCG females were randomly mated with males, while the detection timing of a copulation plug was regarded as gd0.5. The NK cells were suspended in 200 μl of PBS and injected via the tail vein into pregnant females at gd6.5 and gd10.5 (2×10^6^ cells/mouse). Similar amounts of PBS were injected as a control. At gd18.5, the pregnant mice were euthanized. The weight and length of the live fetus and the weight and diameter of the corresponding placenta were recorded from each group.

# Supplementary Figures and Tables

## Supplementary Figures


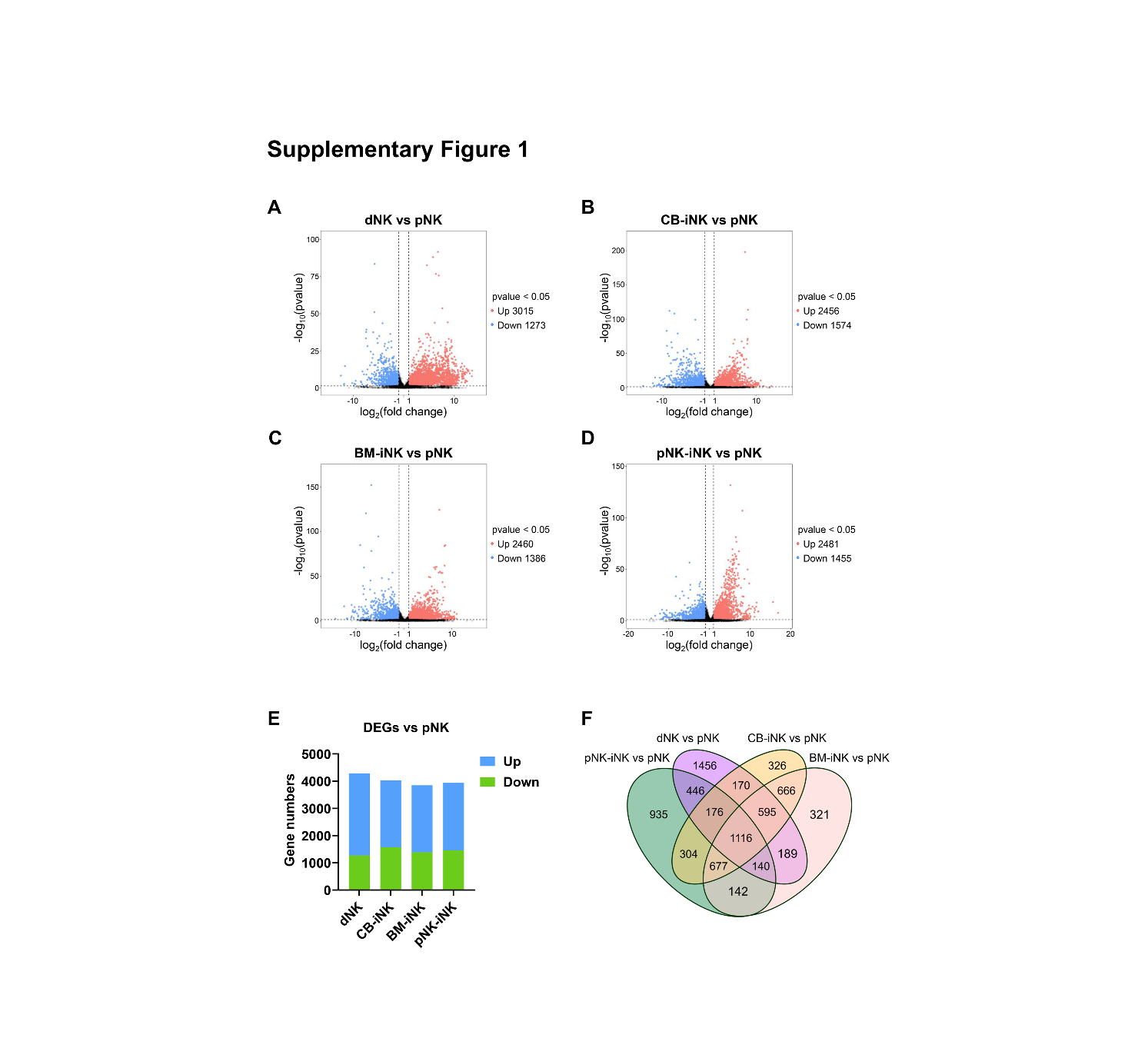


**Supplementary Figure 1.** **Differential gene expression analysis in five kinds of NKs.** **(A-D)** The differentially expressed genes (DEGs), including upregulated genes and downregulated genes compared with pNK are illustrated using a volcano plot: dNK vs pNK(A), CB-iNK vs pNK(B), BM-iNK vs pNK(C), and pNK-iNK vs pNK(D) (fold change > 2 and p-value < 0.05). **(E)** The number of DEGs in each group is shown. **(F)** Venn diagram displaying the overlap of the DEGs is shown in (A-D).

**
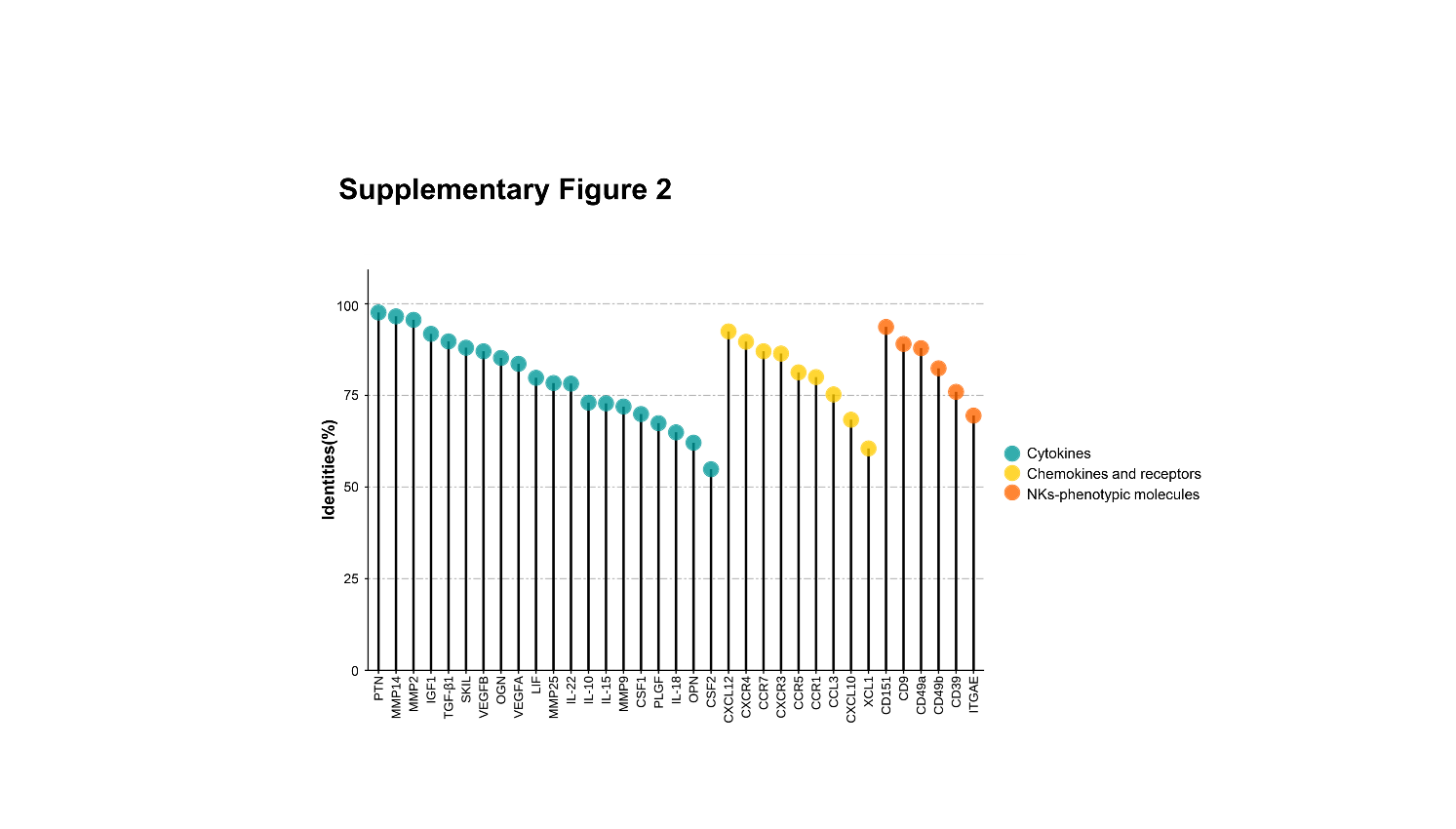
**

**Supplementary Figure 2.** **Phylogenic homology analysis of cytokines, chemokines, chemokines receptors, and phenotypic molecules associated with dNK cells.** Protein sequences were identified using the NCBI HomoloGene system to analyze the distinguishing features between proteins in human NK cells and mice. The results of the identity analysis are presented as a percentage.

**
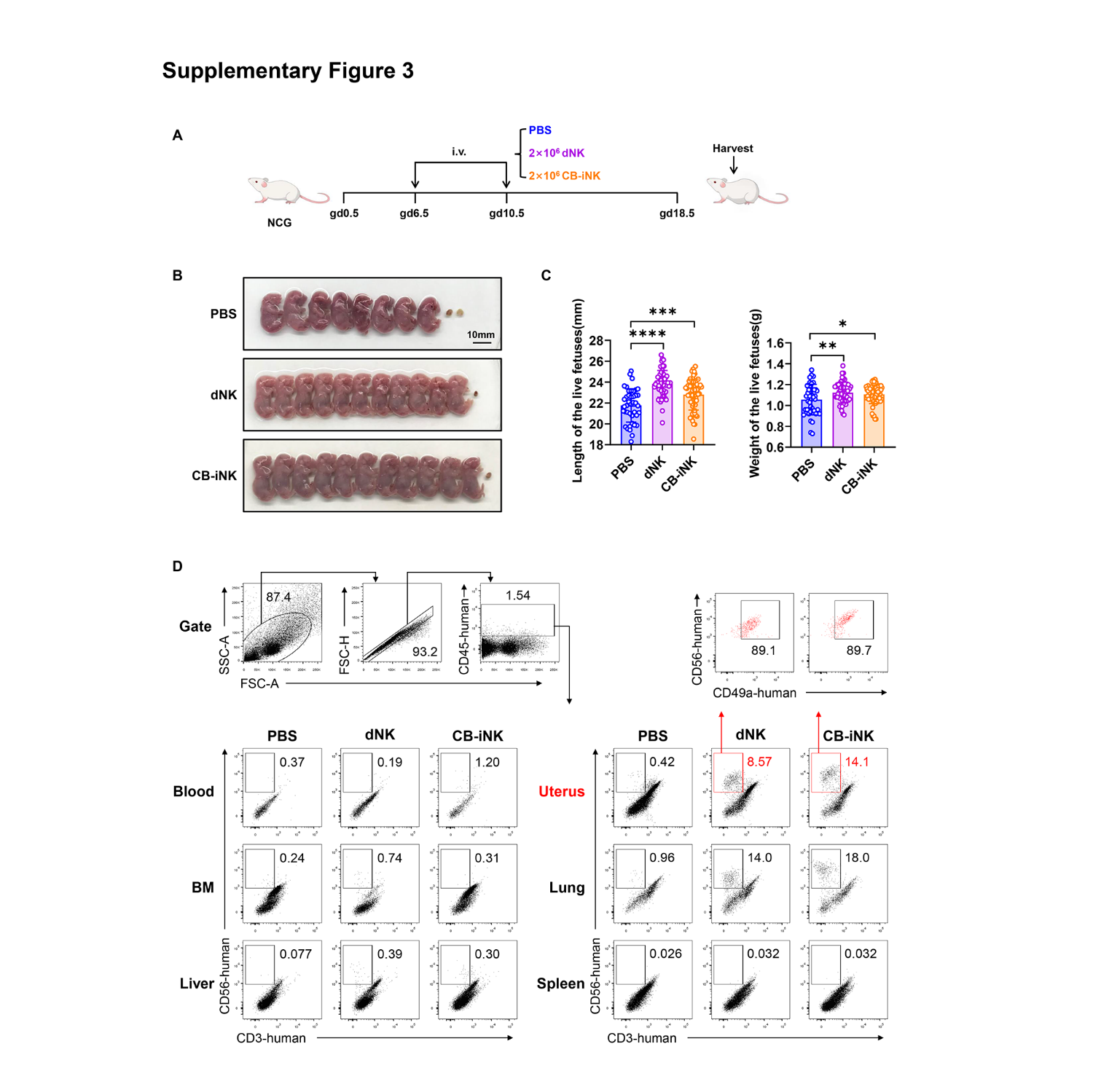
**

**Supplementary Figure 3. Adoptive transferred CB-iNK cells promote fetal growth.** **(A)** Schematic diagram of adoptive transferring CB-iNK cells in pregnant mice, n=6 pregnant mice per group. The experiment was conducted twice, while the results are only shown once. **(B and C)** The pregnancy outcome of adoptive transferring NK cells in mice. **(B)** Representative pictures of fetuses from NK-cells transferred mice and control mice (PBS transferred) at gd18.5. Bar, 10 mm. **(C)** Statistics were calculated by the weight and length of the live fetus in different groups. Data represent means ± SD. Data were analyzed by one-way ANOVA. *p < 0.05; **p < 0.01; ***p < 0.005; ****p< 0.0001. **(D)** The percentage and phenotype of human CD45^+^CD3^-^CD56^+^ NK cells in mononuclear cells of recipient mouse various organs was tested by flow cytometry using anti-human antibodies. The NK cells were suspended in 200 μl of PBS and injected via the tail vein into pregnant females at gd6.5 (2×10^6^ cells/mouse). After 24 h, the pregnant recipient mice were euthanized and the mononuclear cells of the uterus, blood, bone marrow (BM), liver, spleen and lung were examined.

## Supplementary Tables

**Supplementary Table 1.** Antibodies used in this study.

| **Reagent or Resource** | **Sources** | **Identifier** |
| --- | --- | --- |
| FITC Mouse Anti-Human CD3 | BD | Cat# 555332 |
| FITC Mouse Anti-Human CD103 | BD | Cat# 550259 |
| FITC Goat anti-rabbit IgG | BD | Cat# 554020 |
| PE Mouse Anti-Human CD34 | BD | Cat# 555822 |
| PE Mouse Anti-Human CD9 | BD | Cat# 555372 |
| PE Mouse Anti-Human CD151 | BD | Cat# 556057 |
| Alexa Fluor 647 Mouse Anti-Human CD56 | BD | Cat# 557711 |
| Alexa Fluor 647 Mouse anti-Ki-67 | BD | Cat# 561126 |
| APC-Cy7 Mouse Anti-Human CD45 | BD | Cat# 557833 |
| BD Via-Probe Cell Viability Solution (7-AAD) | BD | Cat# 555816 |
| EOMES Monoclonal Antibody (WD1928), PE | eBioscience | Cat# 12-4877-42 |
| PerCP/Cyanine5.5 anti-human CD16 | Biolegend | Cat# 302028 |
| PerCP/Cyanine5.5 anti-human CD3 | Biolegend | Cat# 300328 |
| PerCP/Cyanine5.5 anti-human CD45 | Biolegend | Cat# 368504 |
| PE/Cyanine7 anti-human CD49a Antibody | Biolegend | Cat# 328312 |
| PE/Cyanine7 anti-human CD39 | Biolegend | Cat# 328211 |
| Alexa Fluor 647 anti-human CD49a | Biolegend | Cat# 328310 |
| Brilliant Violet 421 anti-human CD56 | Biolegend | Cat# 318328 |
| Brilliant Violet 605 anti-human CD56 | Biolegend | Cat# 362538 |
| Brilliant Violet 605 anti-human CD3 | Biolegend | Cat# 344836 |
| Zombie Aqua Fixable Viability Kit | Biolegend | Cat# 423101 |
| Human GITR/TNFRSF18 Fluorescein-conjugated Antibody | R&D | Cat# FAB689F |
| Anti-human PTN Purified | LifeSpan | Cat# LS-C162291 |
| Anti-human OGN Purified | LifeSpan | Cat# LS-B10948 |
